# Medial septal GABAergic neurons reduce seizure duration upon optogenetic closed-loop stimulation

**DOI:** 10.1093/brain/awab042

**Published:** 2021-03-26

**Authors:** Katerina Hristova, Cristina Martinez-Gonzalez, Thomas C Watson, Neela K Codadu, Kevan Hashemi, Peter C Kind, Matthew F Nolan, Alfredo Gonzalez-Sulser

**Affiliations:** 1 Centre for Discovery Brain Sciences, Simons Initiative for the Developing Brain, Patrick Wild Centre, University of Edinburgh, Edinburgh, UK; 2 Simons Initiative for the Developing Brain and Patrick Wild Centre, University of Edinburgh, Edinburgh, UK; 3 Open Source Instruments, Watertown MA, USA

**Keywords:** medial septum GABAergic neurons, temporal lobe epilepsy, network stimulation, optogenetics, wireless closed-loop intervention

## Abstract

Seizures can emerge from multiple or large foci in temporal lobe epilepsy, complicating focally targeted strategies such as surgical resection or the modulation of the activity of specific hippocampal neuronal populations through genetic or optogenetic techniques. Here, we evaluate a strategy in which optogenetic activation of medial septal GABAergic neurons, which provide extensive projections throughout the hippocampus, is used to control seizures. We utilized the chronic intrahippocampal kainate mouse model of temporal lobe epilepsy, which results in spontaneous seizures and as is often the case in human patients, presents with hippocampal sclerosis. Medial septal GABAergic neuron populations were immunohistochemically labelled and were not reduced in epileptic conditions. Genetic labelling with mRuby of medial septal GABAergic neuron synaptic puncta and imaging across the rostral to caudal extent of the hippocampus, also indicated an unchanged number of putative synapses in epilepsy. Furthermore, optogenetic stimulation of medial septal GABAergic neurons consistently modulated oscillations across multiple hippocampal locations in control and epileptic conditions. Finally, wireless optogenetic stimulation of medial septal GABAergic neurons, upon electrographic detection of spontaneous hippocampal seizures, resulted in reduced seizure durations. We propose medial septal GABAergic neurons as a novel target for optogenetic control of seizures in temporal lobe epilepsy.

## Teaser

Hristova *et al.* show that medial septal GABAergic neurons, which have dense projections to seizure-generating areas, are structurally and functionally resilient to chronic temporal lobe epilepsy in a mouse model. Stimulating this population with wireless closed-loop optogenetics reduces seizure duration in the mice.

**See Magloire and Lignani (doi:10.1093/brain/awab051) for a scientific commentary on this article.**

## Introduction

New treatment strategies are needed for temporal lobe epilepsy (TLE) as one-third of patients do not achieve seizure control with anti-epileptic drugs.^1–3^ Seizures in TLE can originate from extended or multiple foci and can follow varied propagation patterns throughout the hippocampal formation.^4–6^ Surgical resection is effective in a majority of patients.^7^ However, when it fails to control seizures, it is hypothesized that insufficient tissue is removed.^8^ An alternative option is to modulate the activity of specific brain areas or neuronal populations to block seizures. For example, deep brain stimulation has recently been approved for treating pharmacologically intractable seizures.^9^ Furthermore, techniques that target specific neuronal populations within the hippocampus through genetic manipulation such as overexpression of potassium channels, chemogenetics and optogenetics are able to block seizures or reduce their duration in various TLE animal models and could be translated to the clinic.^10–14^ However, directly targeting cellular populations across the hippocampal formation, with its bilateral organization and large volume, may not be the most effective strategy if only a small component of the seizure foci is controlled. A potential alternative approach to treating TLE is to target neuronal populations that can powerfully modulate the activity across the larger epileptogenic network.

Medial septal GABAergic neurons (MSGNs) may be a suitable population for stimulation to block seizures. MSGNs send extensive projections across the hippocampal formation and target GABAergic cells in structures critical for seizure initiation and propagation such as the hilus in the dentate gyrus, the subiculum and the medial entorhinal cortex.^11^^,^^14–18^ MSGNs are necessary for normal oscillatory activity in the hippocampus and their activation can modulate hippocampal network rhythms.^19–23^

The medial septum also receives direct inputs from the hippocampus, it is one of the first structures to which seizures spread to in TLE models and its activity is correlated with that of the hippocampus in physiological and epileptic conditions.^24–27^ Furthermore, the medial septum itself is a small midline area that can be easily targeted for modulation with techniques such as deep brain stimulation or gene therapy.

A recent study suggests that cholinergic medial septal neuron stimulation reduces seizure activity via excitation of hippocampal somatostatin GABAergic neurons, while targeting MSGNs appears ineffective in a kindling model where seizures are generated in response to electrical stimulation.^28^

Here, we evaluated the feasibility of optogenetic stimulation of MSGNs to stop seizures in a chronic TLE model which closely approximates the disease, as pathological hippocampal sclerosis develops, and seizures resistant to several anti-epileptic drugs occur spontaneously.^29^^,^^30^ We tested a transient stimulation strategy where stimulation occurs in response to a computer-detected seizure, that is likely to have less adverse effects than continuous stimulation. We found that MSGNs and their connections are maintained in this model. We show that optogenetic stimulation of MSGNs can effectively modulate local field potential (LFP) activity across the hippocampal network in conditions of chronic epilepsy and does not negatively affect ongoing behaviour. We then developed a technique for chronic wireless optogenetics and electrophysiology that allowed us to stimulate MSGNs upon detection of spontaneous hippocampal seizures. We found that wireless closed-loop stimulation of MSGNs decreased seizure durations. Together, our results suggest that optogenetic stimulation of MSGNs may be a feasible strategy for suppression of currently intractable seizures.

## Materials and methods

### Animals

All animal procedures were undertaken in accordance with the University of Edinburgh animal welfare committee regulations and were performed under a UK Home Office project license. Six to 18-week-old male and female *VGAT-IRES-Cre* mice [strain name: Slc32a1^tm2(cre)Lowl^, Jackson Labs; stock number: 028862] were crossed with C57Bl6J (RRID:IMSR_JAX:000664) mice to maintain the line heterozygous at the transgene insertion locus.

### Viral injection and surgery

Mice were anaesthetized with isoflurane and mounted on a stereotaxic frame (David Kopf Instruments). Adeno-associated virus (AAV) expressing either mRuby conjugated to synaptophysin and membrane-bound green fluorescent protein (GFP) under the control of the synapsin promotor (AAV-hSyn-Flex-mGFP-2A-Synaptophysin-mRuby, Addgene plasmid 71760, serotype 1/2, packaged into AAV),^31^ channelrhodopsin-2 (ChR2) conjugated to mCherry [AAV-EF1a-DIO-hChR2(H134R)-mCherry-WPRE-pA, serotype 5, Addgene plasmid 20297 purchased from University of North Carolina Vector Core, USA] or mCherry (AAV-EF1a-fDIO-mCherry, serotype 5, Addgene plasmid 114471, purchased from University of North Carolina Vector Core, USA) was injected through a craniotomy (0.6 mm rostral, 0.0 mm caudal to bregma). Two injections of 450 nl were made (3.4 and 3.2 mm ventral from the brain surface).

A guide cannula (polar fused silica tubing length = 10 mm, Ø = 0.32 mm, Sigma-Aldrich) for later kainate injection was implanted over the left hippocampus (1.9 mm caudal, 1.2 mm lateral from bregma and 1.4 mm ventral from the brain surface).

### Surgery for tethered optogenetic stimulation and multisite recordings

After viral injection and cannula placement, an optical fibre (PlexBright Fibre Stub, length = 13 mm, Ø = 200/230, 0.66NA, Plexon) was implanted (0.6 mm rostral, 0.2 mm lateral from bregma and 2.6 mm ventral at a 4.5° angle from the brain surface) over the medial septum. Pairs of local LFP electrodes (Ø = 50.8 μm, Teflon insulated stainless steel, A-M systems) were implanted targeting the molecular layer of the dentate gyrus in five locations across the rostral-to-caudal extent of the hippocampus (contralateral to implanted cannula: 1.85 mm caudal, 1.25 mm lateral from bregma and 1.40 mm ventral from brain surface; bilaterally: 2.3 mm caudal, 1.8 mm lateral from bregma and 2.0 mm ventral from the brain surface; bilaterally: 3.3 mm caudal, 3.3 mm lateral from bregma and 2.9 mm ventral from the brain surface). Two miniature ground screws (Yahata Neji, M1 Pan Head Stainless Steel Cross, RS Components) were attached over the cerebellum (5.0 mm caudal, 2 mm lateral) to serve as ground as well as three additional screws for structural support. The electrodes were attached to an electronic interface board (EIB-16, Neuralynx). The cannula, optical fibre and electrode assemblies were fixed to the skull using a combination of UV activated cement (3M Relyx Unicem 2 Automix, Henry Schein) and dental cement (Simplex Rapid, Kemdent).

### Surgery for wireless optogenetic stimulation and hippocampal seizure monitoring

After viral injection and cannula placement, a wireless optogenetic device was implanted.^32^ The main body of the device, consisting of a ∼9.8 mm diameter circular conductive receiver and surface-mounted capacitor and rectifier to power the LED when located in an inductive field, was placed on the skull. A micro-LED at the injectable needle tip of the device (470 nm emission wavelength, needle length = 4 mm, LED dimensions in micrometres: 270 × 220 × 50, Neurolux) was implanted lateral to the medial septum (0.6 mm RC, 0.15 mm lateral to bregma and 3.3 mm ventral from the brain surface). A battery-powered single-channel electrophysiology transmitter (A3028B, Open Source Instruments) was implanted subcutaneously on the back of the mouse and the signal and ground leads were tunnelled under the skin to the skull. The signal lead was connected to an LFP electrode (Ø = 127 μm, Teflon insulated platinum-iridium, Science Products) targeting the molecular layer of the dentate gyrus implanted ipsilaterally to the cannula at an intermediate rostral to caudal location (2.3 mm caudal, 1.8 mm lateral and 2.0 mm ventral from the brain surface). The ground lead was placed on the cortical surface in the contralateral hemisphere (3.2 mm caudal, 3.0 mm lateral and 0.1 mm ventral from the brain surface) and held in place by a miniature screw (Yahata Neji, M1 Pan Head Stainless Steel Cross, RS Components). Two additional screws were placed for structural support. The cannula, wireless optical device and electrode assemblies were fixed to the skull using a combination of UV activated cement (3M Relyx Unicem 2 Automix, Henry Schein) and dental cement (Simplex Rapid, Kemdent).

### Seizure induction

Mice were allowed to recover from surgery for at least 1 week before induction of status epilepticus, which leads to hippocampal sclerosis and chronic spontaneous seizures after ∼2 weeks.^30^ Mice were anaesthetized with isoflurane and were injected with 1 ml of 5% dextrose saline, to prevent dehydration during status epilepticus. Kainate (100 nl, 20 mM in saline, Tocris) was infused into the left dorsal hippocampus targeting the molecular layer of the dentate gyrus, via an injection cannula (internal cannula with 0.2 mm projection for a 1.6 mm ventral from the brain surface, PlasticsOne), through the previously implanted guide cannula resulting in status epilepticus. Chronic seizure manifestation was not confirmed in mice to be utilized solely for anatomical analyses or in experiments to test functionality of MSGN optical stimulation to entrain hippocampal-wide oscillations. However, behavioural manifestations of status epilepticus upon kainate injection and hippocampal sclerosis had to be present for inclusion in the study.

### Immunohistochemistry and imaging

Mice were anaesthetized with isoflurane followed by a lethal dose of sodium pentobarbital and transcardially perfused with phosphate-buffered saline (PBS; Invitrogen) followed by 4% paraformaldehyde (PFA; Sigma Aldrich) in 0.1 M phosphate buffer (Sigma Aldrich). Brains were removed and post-fixed overnight in 4% PFA, then rinsed in PBS and incubated overnight in 30% sucrose in PBS. Tissue was then placed in Optimum Cutting Temperature (OCT) embedding matrix and sliced coronally in 60-µm thick sections using a freezing vibratome. Free-floating sections of the entire medial septum and hippocampus were collected and stored in PBS with sodium azide 0.05% (Sigma Aldrich) at 4°C until used.

Sections were rinsed in PBS, then permeabilized with 0.3% Triton^TM^ X-100 (Sigma-Aldrich) in PBS (PBST). Selected anatomical levels of the hippocampus were incubated overnight in Neurotrace (1:500; 640/660 or 500/525 or 400/450; Life Technologies) in PBST at 4°C. Selected anatomical levels of the medial septum were incubated overnight in primary antibodies mixed in PBST at 4°C (Supplementary Table 1), sections were then rinsed and incubated in secondary antibodies mixed in PBST overnight at 4°C (Supplementary Table 2). Finally, sections were rinsed several times in PBS and mounted onto slides.

Confocal images for fluorescence were taken with a Nikon A1 or a Zeiss LSM800 confocal. For medial septal analysis, three coronal levels at 0.85, 0.7 and 0.5 mm rostral to bregma were imaged. Stacks of images (24 µm, 2 µm *z*-steps) containing the medial septum were acquired using a 20× Plan Apo VC DIC N2 objective. For hippocampal synaptophysin puncta analysis, four anatomical levels caudal to bregma were selected at 1.82 mm, 2.3 mm, 2.85 mm and 3.28 mm and six images (1 µm optical slice) at each level were taken from medial and lateral CA1 in stratum oriens and strata radiatum/lacunosum moleculare, CA3 stratum radiatum and the hilus within the dentate gyrus at each plane (Supplementary Fig. 2). Images were acquired using a Plan Apo 40× oil DIC H objective.

To evaluate AAV axonal expression and the anatomical location of electrodes, optical devices and cannulas in electrophysiological experiments, tiled fluorescent images were acquired across all brain slices containing the medial septum and hippocampus using a Zeiss Axio Scan.Z1 microscope and a Plan-Apochromat 10×/0.45 M27 objective. Only mice expressing fluorophores bilaterally within the medial septum and displaying hippocampal sclerosis were included in further analyses.

For histological analysis, researchers were blinded to treatment. Quantification of medial septum virus expression and immunolabelled neurons was performed with FIJI-ImageJ (NIH). Synaptic puncta were automatically counted with Imaris (Oxford Instruments) with the Spots module by setting an automated threshold at ∼0.77 µm. Puncta counts were normalized to the number of fluorophore-expressing cells in the medial septum and the area imaged at each level (159.1 µm^2^ for the hilus and 954.6 µm^2^ for the hippocampus as a whole).

Contrast and brightness for images in figures was adjusted with FIJI-ImageJ (NIH).

### Multisite tethered recordings and optogenetic stimulation

Mice were placed in 50 × 50 cm square arenas and connected for recordings to an RHD 16-channel recording headstage (Intantech) through an electrical commutator (Adafruit) and an acquisition board (OpenEphys). LFP signals were sampled at 1 kHz and referenced to ground. Mice were connected to a fibre-coupled LED (blue = 465 nm, Plexon) via optical patch cords which directed the light to a 1 mm optical ferrule (Plexon) and the ceramic sleeve of the previously surgically implanted optical fibre. The power of the LED was calibrated to emit an irradiance at the implanted fibre stub tips of ∼12.7 to 31.9 mW/mm^2^. One hundred and twenty epochs of 10-ms long square pulses at 10 Hz were applied for 30 s with an interval of 2 min between epochs in both non-epileptic and epileptic conditions utilizing a Master-8 (AMPI). Mice were video-recorded during stimulation sessions at 10 frames/s (C270 HD webcam, Logitech).

Quantification of LFP entrainment upon MSGN optical stimulation was performed by calculating phase locking values (PLVs), the phase-angle difference clustering in polar space across trials. Analysis was performed utilizing custom-made Python scripts. As wiring failure during surgery occurred in some leads, traces from all electrodes were checked visually and electrodes with an absent signal were discarded. LFP traces from electrode pairs at individual hippocampal locations were visually identical. Therefore, when both electrodes were available, the one used for analysis was picked randomly. To extract phase angle information across 30 s stimulation and prestimulation baseline data across all frequency bands, the Hilbert transform was applied to LED and LFP channel voltage traces using the *apply_hilbert* function from the Python MNE toolbox. Phase angles were then calculated using the SciPy *angle* function and differences between the LED and individual LFP electrodes were calculated using the following equation:

(1) $$\left|n^{-1}\sum_{t=1}^{n}e^{i\left(\emptyset_{\mathrm{LED}\left(t\right)}-\emptyset_{\mathrm{Electrode}\left(t\right)}\right)}\right|$$

in which *n* is the number of time points, *t* is the trial number and Ø_LED_ and Ø_Electrode_ are phase angles from the LED and analysed electrode.^33^^,^^34^ Phase angle differences were then multiplied by the imaginary operator and averaged per time point across trials. The PLV mean value was obtained by calculating the average absolute phase angle difference value across all trial-averaged epoch time points. Mean PLV baseline values were then subtracted from stimulation epochs for statistical comparison.

Power spectral density (PSD) was calculated for each 30 s baseline and stimulation LFP using the SciPy Python function *Periodogram.*^35^ Entrainment of the signal to the 10 Hz stimulation was quantified as the ratio of the cumulative PSD around the optical stimulation frequency (±1 Hz) to the cumulative PSD in the 3 to 13 Hz band.^19^

Quantification of behaviour during optical stimulation in multisite tethered recordings was performed *post hoc* through manual analysis of videos. Concurrent LFP analysis was used to ascertain whether animals were asleep when lack of movement was detected. For trials when animals were not moving, 5 s of LFP prestimulation trials were plotted and visually assessed. Animals were classified as awake if the presence of low amplitude LFP activity was detected, or classed as asleep if high amplitude low-frequency (<3 Hz) oscillations (non-REM sleep) or theta frequency (4–12 Hz) oscillations (REM sleep) were present.^36^ Behaviour was viewed as the action an animal was engaged in at the start of and throughout optical stimulation, including grooming, eating, exploring, quiet rest or sleep. When the action of the animal did not change throughout the trial, the continuous action was assessed for changes in speed.

Analyses were performed blinded to virus injected. Only mice expressing fluorophores bilaterally within the medial septum and displaying hippocampal sclerosis were included in analyses. The analysis code is available at: https://github.com/Gonzalez-Sulser-Team/Entrainment-Analysis.

### MSGN closed-loop optogenetic stimulation to modulate seizure duration

We injected mice with kainate 1 week after the initial surgery and we began seizure detection at least 2 weeks after injection, to allow for the establishment of chronic spontaneous seizures and hippocampal sclerosis, which we confirmed anatomically *post hoc*. At least 2 weeks after kainate injection, mice were placed in a home cage installed with loop induction antennas connected to a tuner box and a power distribution control box (Neurolux), to inductively power the previously surgically implanted wireless optogenetic devices upon seizure detection. The home cage and optical stimulation equipment were placed within an FE2F Faraday enclosure (Open Source Instruments) adjacent to LFP receiver antennas connected externally to an octal data receiver, LWDAQ driver (Open Source Instruments) and a recording computer. Continuous LFP signals (512 Hz acquisition rate, LWDAQ software, Open Source Instruments) and video at (10 frames/s, C270 HD webcam, Logitech) were recorded for a single mouse at a time for 1–2 weeks depending on wireless electrophysiology transmitter battery. LFP signals were analysed in real-time by a PC running a custom-made LWDAQ seizure detection algorithm to determine the presence of spontaneous seizures (see below). When the required criteria were met, the detection algorithm time-stamped a seizure for later review and in 50% of seizures (randomized) triggered the activation of the wireless LED device implanted in the mouse, via a TTL pulse from the octal data receiver to the power distribution control box, resulting in 30 s of stimulation of 10 ms square pulses at 10 Hz at an estimated irradiance of ∼5 mW/mm^2^. Electrophysiological seizure durations were analysed off-line by trained experimenters blinded to LED status and virus injected. Only mice expressing fluorophores bilaterally within the medial septum, hippocampal sclerosis and detected electrographic seizures were included in the analyses.

Behavioural seizures were scored utilizing a modified six-point Racine’s scale^37^: 1 = mouth or facial automatisms; 2 = two or less myoclonic jerks; 3 = three or more myoclonic jerks and/or forelimb clonus; 4 = tonic-clonic forelimb and back extension; 5 = tonic-clonic forelimb and back extension with rearing and collapsing; and 6 = tonic-clonic forelimb and back extension with wild running or jumping.

### Online electrographic seizure identification

Data were recorded and analysed online in 1-s time intervals and compared to a library of previously recorded seizures from an initial cohort of mice (*n*** **=** **4) with spontaneous chronic seizures 2 weeks after intrahippocampal kainate using the following measurements: (i) coastline: the sum of the absolute changes in voltage values in an interval; (ii) intermittency: the fraction of the coastline generated by the 10% largest steps in an interval; (iii) spikiness: the ratio of the maximum voltage range across all 19.6-ms bins in a 1-s time interval to the median range value across the entire interval; and (iv) coherence: the fraction of the voltage area under the curve occupied by the 10 largest peaks and trough pairs in an interval. Measurements were then converted into bounded sigmoidal values and compared in real time with a library of previously recorded seizures. If a threshold of similarity of 0.1 across all metrics was crossed, an interval was classified as a seizure. Three consecutive seizure intervals resulted in a seizure timestamp resulting in random activation of the optical device in 50% of seizures. The code and further details about the analysis are available at: http://www.opensourceinstruments.com/Electronics/A3018/Seizure_Detection.html#Closed%20Loop%20with%20ECP20.

### Statistical analysis

Pilot experiments were performed on three to four animals to establish a rationale for the sample size. All statistical analyses were performed using OriginPro software. Normality of groups was assessed with the Shapiro-Wilk test. The anatomical effects of kainate compared to saline on medial septal neuronal populations and MSGN projections to the hippocampus were compared using a two-way ANOVA with a Tukey *post hoc* test. Comparisons of mean PLVs and median entrainment efficiency of mCherry-ChR2 with mCherry control mice in pre-epileptic conditions across electrodes were performed with a two-way ANOVA with a Tukey *post hoc* test. Control and epileptic conditions in mCherry-ChR2 expressing mice and onset delays across electrodes in control and epileptic conditions were compared with a repeated measures two-way ANOVA with a Tukey *post hoc* test. The distribution of seizure durations and the distribution of stimulation epochs in light off and light on conditions in individual mice were compared using a Kolmogorov-Smirnov test. Median seizure duration distributions across all seizures in mCherry-ChR2 or mCherry control expressing mice in light off and light on conditions and, median interseizure intervals were compared with a paired Wilcoxon signed-rank test. Comparisons of percent light off and light on epochs with behavioural changes and comparison of normalized median seizure duration changes between light off and light on between mCherry-ChR2 and mCherry control expressing mice, were performed using two-sample *t*-tests. Median behavioural seizure severity was compared with a paired *t*-test.

### Data availability

All Python and LWDAQ scripts are freely available. The data that support the findings of this study are available from the corresponding author, upon reasonable request.

## Results

### Anatomical assessment of MSGNs and their projections in chronic TLE with hippocampal sclerosis

**Figure 1:**
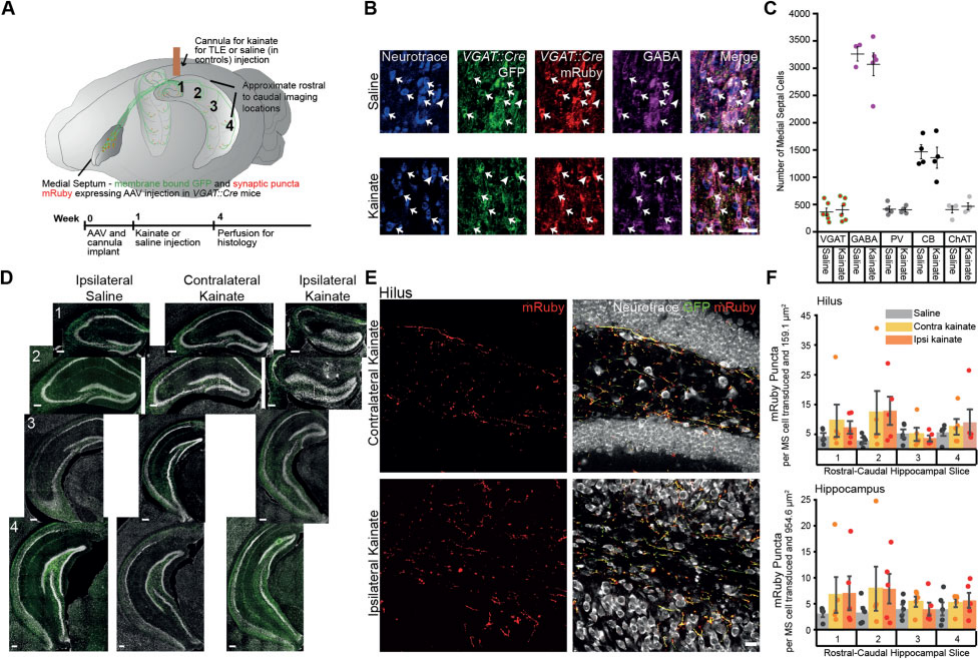
**MSGNs and their connections across the hippocampus remain despite hippocampal sclerosis.** (**A**) *Top*: Schematic of viral expression and cannula placement. Cre-dependent GFP and mRuby conjugated to synaptophsyin were expressed in MSGNs by AAV injection into medial septum of *VGAT::cre* mice. A cannula was implanted for kainate or vehicle (saline) unilateral injection over rostral-dorsal hippocampus. Numbers correspond to approximate hippocampal rostral-caudal levels imaged for puncta analysis. *Bottom*: Experimental timeline. (**B**) Representative GFP-mRuby AAV expression in MSGNs and staining for Neurotrace and GABA in saline and kainate treated mice. Scale bar = 50 µm. Examples of neurons expressing GFP, mRuby or GABA (arrows) and neurons not expressing GFP, mRuby or GABA (arrowheads). We found 96.16 ± 6.46% of mRuby-GFP expressing cells co-expressed GABA. (**C**) Neuronal populations in saline and kainate treated mice. Horizontal lines indicate mean values [mean ± standard error of the mean (SEM)]. Points correspond to values from individual mice. Populations were not significantly decreased in kainate-treated mice when compared to saline-treated controls (two-way ANOVA, *P *=* *0.58, F = 0.31, df = 1, *n* of saline and kainate treated mice per cell type, respectively: mRuby-GFP labelled cells in *VGAT::Cre* mice *n* = 6, 7; GABA *n* = 3, 5; PV, CB, ChAT *n* = 4, 4). (**D**) Representative hippocampal sections of rostral-caudal levels stained with fluorescent Neurotrace are shown in a saline-injected mouse, and the contralateral and ipsilateral hippocampi of a kainate-injected mouse. Scale bars = 200 µm. Expression of GFP (green) in MSGN axons across the hippocampus and sclerosis in rostral slices ipsilateral to kainate injection. (**E**) Putative synaptic terminals expressing mRuby in the hilus at second rostral-caudal level contralateral and ipsilateral to kainate injection (dashed white boxes in **D**). Scale bar = 10 µm. (**F**) Density of synaptic terminals across rostral-caudal levels in the hilus and the entire hippocampus in saline-treated mice and contralateral and ipsilateral hippocampi of kainate-treated mice. Bars indicate mean (mean ± SEM). Points correspond to values from individual mice. Synaptic density did not decrease in kainate-treated mice (two-way ANOVA, *P *=* *0.57, F = 0.87, df = 11, *n *=* *5 mice per treatment). Puncta counts were reported normalized to the number of virus-labelled cells in medial septum.

We first determined if MSGNs can be specifically labelled using transgenic mice in which Cre expression is controlled by the promoter of the vesicular GABAergic transporter (*VGAT::Cre*) in combination with injected AAVs expressing Cre-dependent transgenes. We injected a Cre-dependent AAV encoding mRuby conjuated to synaptophysin, to allow us to image putative synaptic puncta, and membrane-bound GFP,^38^ into the medial septum of *VGAT::Cre* mice (Fig. 1A and B). We found that cell bodies in the medial septum that express virally delivered mRuby and GFP were also labelled with immunohistochemical markers of MSGN subpopulations^15^^,^^20^^,^^39^^,^^40^ including GABA, parvalbumin (PV) and calbindin (CB) (*n*** **=** **3 mice; Fig. 1B and Supplementary Fig. 1D). Neurons that expressed the virally delivered markers were not co-labelled with antibodies against choline acetyl transferase (ChAT), which labels cholinergic neurons in the medial septum (*n*** **=** **3 mice; Supplementary Fig. 1D).

We assessed the susceptibility of MSGNs to hippocampal sclerosis in the intrahippocampal kainate TLE model. After viral injection into the medial septum, we implanted mice with a cannula over the hippocampus and injected kainate 1 week after surgery to induce seizures. Three weeks after seizure induction, and consistent with previous studies,^30^^,^^41^^,^^42^ we observed hippocampal sclerosis and expansion of the dentate gyrus granule cell layer (Fig. 1D and E).^30^^,^^42^ We found that there was no reduction in the number of MSGNs expressing VGAT (mRuby-GFP expressing cells in AAV injected *VGAT::Cre* mice) or immunohistochemically labelled GABA, PV and CB, or in the number of ChAT-expressing cells when compared to saline injected controls (two-way ANOVA with a Tukey *post hoc* test, *P*** **=** **0.58, F = 0.31, df = 1, *n* of saline and kainate treated mice per cell type, respectively: mRuby-GFP labelled cells in *VGAT::Cre* mice *n* = 6, 7 GABA *n* = 3, 5; PV, CB, ChAT *n* = 4, 4) (Fig. 1B, C and Supplementary Fig. 1C). Therefore, MSGNS are structurally resilient to kainate-induced hippocampal sclerosis.

We tested if putative synaptic connections from MSGNs to the hippocampus are reduced in TLE with hippocampal sclerosis. We imaged across the rostral to caudal axis of the hippocampus and found that MSGN GFP-labelled axons and mRuby puncta marking putative pre-synapses accumulated in or close to the pyramidal and granule cell layers, lacunosum moleculare and in the hilus of the dentate gyrus, areas where hippocampal GABAergic cell bodies are located (Fig. 1D, E and Supplementary Fig. 2A and B).^43^ Mice received a unilateral injection of kainate to induce chronic seizures and hippocampal sclerosis, or saline, in controls, and were sacrificed 21 days after injection to perform histological analysis (Fig. 1A). We found no significant reduction in the number of putative synapses from MSGNs when comparing both ipsilateral and contralateral hippocampi in kainate-treated animals to ipsilateral hippocampi in controls, across the rostral to caudal extent of both the hilus in the dentate gyrus (two-way ANOVA, *P*** **=** **0.57 F = 0.87, df = 11, *n*** **=** **5 saline and 5 kainate treated mice; Fig. 1E and F), an area critical for seizure propagation,^11^^,^^44–46^ and the hippocampus as a whole (two-way ANOVA, *P*** **=** **0.79, F = 0.63, df = 11, *n*** **=** **5 saline and 5 kainate treated mice) (Fig. 1F and Supplementary Fig. 2B). The overall survival of putative synapses indicates that MSGN stimulation may be capable of influencing hippocampal oscillatory activity in TLE.

### Hippocampal-wide LFP modulation by MSGN optogenetic stimulation in TLE with hippocampal sclerosis

To test whether MSGN hippocampal projections remain functional in epileptic conditions with hippocampal sclerosis, we determined whether MSGN optogenetic stimulation can modulate oscillatory activity bilaterally across the rostral to caudal extent of the hippocampus. We injected AAV encoding channelrhodopsin-2 fused to mCherry (ChR2-mCherry) or, in controls, encoding only mCherry in the medial septum of *VGAT::Cre* mice. We found that over 90% of cell bodies expressing virally delivered mCherry co-labelled with GABA in animals injected with AAV encoding ChR2-mCherry or mCherry only (*n*** **=** **3 mice; Supplementary Fig. 1D).

**Figure 2:**
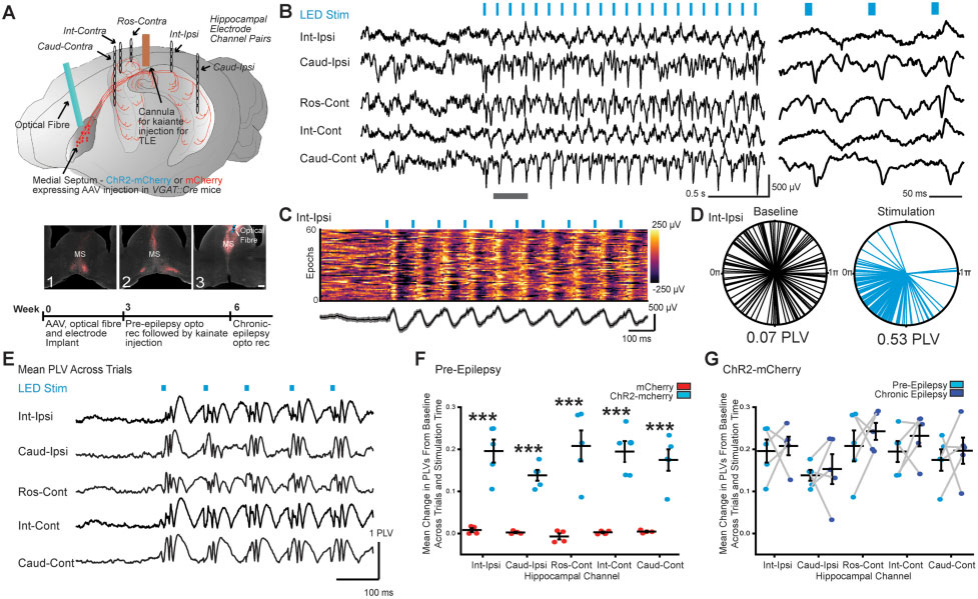
**Entrainment of oscillations across the hippocampus by optical stimulation of MSGNs despite hippocampal sclerosis.** (**A**) *Top*: Schematic of viral expression and implantation of optical fibre, electrodes and cannula. Cre-dependent ChR2-mCherry or mCherry were expressed in MSGNs by AAV injection into medial septum of *VGAT::cre* mice. An optical fibre for MSGN stimulation, a cannula for kainate injection and pairs of electrodes for tethered recordings rostral contralateral to cannula (Ros-Cont) and bilaterally at intermediate (Int-Ipsi and Int-Cont) and caudal (Caud-Ipsi and Caud-Cont) locations were implanted. *Bottom*: ChR2-mCherry expressed in the medial septum in rostral to caudal slices (1–3) with optical fibre track. Scale bar = 100 µm. (**B**) Representative LFP traces in a chronically epileptic mouse from hippocampal channels before and after onset of 10 Hz theta optical MSGN stimulation (*left*) and expanded time view over grey bar in *left* panel (*right*). (**C**) *Top*: Colour-coded voltage traces for 60 example consecutive epochs. *Bottom*: Average (black line) and standard deviation (grey) of example epochs. (**D**) Example polar-plot of LED-LFP phase-angle differences across trials (individual lines) at one sampling time point, 35 ms after start of baseline or stimulation epochs from one mouse. Mean PLVs calculated from clustering of phase-angle differences across trials are indicated. (**E**) PLVs over time averaged across trials before and during stimulation for all electrodes in example mouse. (**F**) Plot of baseline-subtracted mean PLVs across all stimulation times and epochs in mice expressing mCherry or ChR2-mCherry in MSGNs. Horizontal lines indicate mean values (mean ± SEM). Points correspond to mean values from individual mice. The PLV was significantly higher across all electrodes in ChR2-mCherry expressing mice when compared to mCherry controls (***P <* *0.0001; two-way ANOVA, Tukey *post hoc* test, *n *=* *5 mice per treatment). (**G**) Plot of baseline-subtracted mean PLVs across all times and epochs per electrode in conditions preceding and 21 days after kainate injection in ChR2-mCherry expressing mice. Horizontal lines indicate mean values (mean ± SEM). Points correspond to mean values from individual mice. Hippocampal sclerosis did not diminish the capacity of MSGN optical stimulation to entrain hippocampal oscillations (two-way ANOVA repeated measures, *P *>* *0.05, *n *=* *5 mice).

In experimental animals, seizures were induced by delivery of kainate through a cannula targeting the dorsal hippocampus. To enable activation of ChR2-expressing neurons, we implanted an optical fibre over the medial septum. To record hippocampal LFP activity we implanted electrodes in five locations in the molecular layer of the dentate gyrus, an area critical for gating the spread of seizures^11^^,^^44–46^; one location was contralateral to the cannula at the same rostral-to-caudal level and two additional locations were ipsi- and contralateral to the cannula at progressively more ventral locations (Fig. 2A, see Supplementary Fig. 3 for confirmed optical fibre and electrode histological locations). Three weeks after surgery, to allow for viral expression, mice were connected to tethered amplifiers and LEDs and were placed in square arenas for recordings.

To test whether we could modulate network oscillations across the hippocampal formation in non-epileptic conditions, we stimulated MSGNs prior to seizure induction. We used a stimulation frequency of 10 Hz, which is in the range of normally occurring theta oscillations and LFP spiking activity during seizures. We performed 10 Hz optical stimulation for 30 s with a 90-s interval between epochs. In mice injected with AAV encoding ChR2-mCherry, the onset of stimulation produced a shift in LFP oscillations at all recording locations that matched the 10 Hz stimulation frequency and was consistent across epochs (Fig. 2B, C and Supplementary Fig. 4).

To quantify the effect of rhythmic MSGN stimulation upon hippocampal activity we compared phase locking statistics between mice expressing ChR2 in MSGNs with control mice expressing mCherry. We calculated the PLVs of each LFP trace to LED stimulation at every sampling time point across trials in baseline and stimulation periods; the PLV metric approaches 1 when there is little phase difference and 0 if the signals are unrelated at each time point across trials (Fig. 2D).^33^^,^^34^ Trial-averaged PLVs increased after LED stimulation across all channels (Fig. 2E). The average baseline-subtracted PLV across trials and all trial sampling time points was significantly higher across all electrode locations in mice expressing ChR2-mCherry when compared to mCherry expressing control mice (two-way ANOVA, Tukey *post hoc* test, *P*** **=** **0.002, 0.002, 0.0006, 0.009, 0.0004 for intermediate-ipsilateral, caudal-ipsilateral, rostral-contralateral, intermediate-contralateral, caudal-contralateral electrode locations, respectively, df = 4, F = 0.18, *n*** **=** **120 trials per mouse, *n*** **=** **5 mice, Fig. 2F).

**Figure 3:**
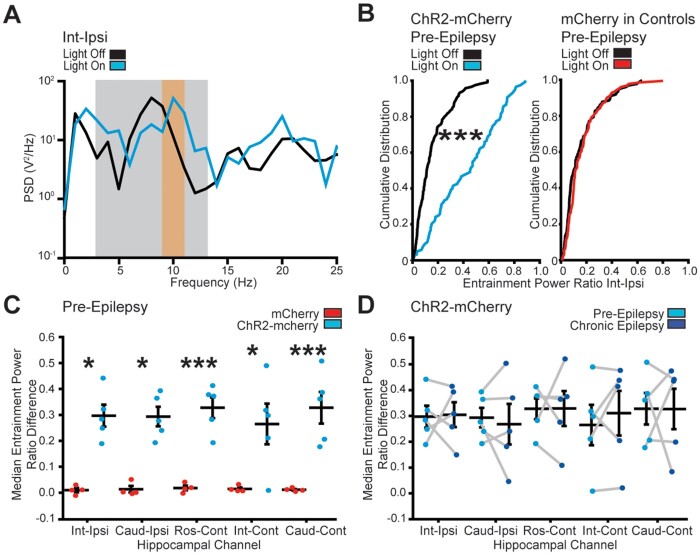
**Entrainment ratio analysis of hippocampal oscillations during MSGN stimulation.** (**A**) Example power spectral density (PSD) plot displaying LFP power plotted against frequency during a baseline—light off epoch (black line) and a stimulation—light on epoch (blue). Note, entrainment ratio is calculated by dividing the cumulative power in the stimulation range (orange bar) by the cumulative power at the extended theta range (grey bar). (**B**) Example cumulative probability distributions of entrainment power ratio at the intermediate ipsilateral electrode across all epochs in individual mice expressing ChR2-mCherry (*left*) or mCherry only (*right*) in pre-epileptic conditions. The amplitude ratios were significantly increased during optogenetic stimulation in all mice expressing ChR2-mCherry, but not in mCherry expressing controls (Kolmogorov-Smirnov Test, *n *=* *120 stimulation epochs per mouse, ***P <* *0.0001). (**C**) Plot of the median entrainment power ratio difference between light on and light off trials per electrode in mice expressing mCherry or ChR2-mCherry. Horizontal lines indicate mean values (mean ± SEM). Points correspond to median values from individual mice. The entrainment efficiency was significantly higher across all electrodes in ChR2-mCherry expressing mice when compared to mCherry expressing mice (two-way ANOVA, Tukey *post hoc* test, *P *=* *0.013, 0.020, 0.008, 0.049, 0.005 for intermediate-ipsilateral, caudal-ipsilateral, rostral-contralateral, intermediate-contralateral, caudal-contralateral electrode locations, respectively, df = 4, F = 0.18, *n *=* *120 trials per mouse, *n *=* *5 mice) *P <* *0.05; ***P <* *0.0001. (**D**) Plot of the median entrainment power ratio difference between light on and light off trials per electrode in conditions preceding and 21 days after kainate in ChR2-mCherry expressing mice. Horizontal lines indicate mean values (mean ± SEM). Points correspond to median values from individual mice. Chronic seizures did not diminish the capacity of MSGN optical stimulation to entrain oscillations in the hippocampus (two-way repeated measures ANOVA, df = 4, F = 0.01, *P *=* *0.91 Tukey *post hoc* test, *n *=* *120 baseline and stimulation epochs per condition, *n *=* *5 mice).

To evaluate entrainment of hippocampal LFPs by MSGN stimulation we calculated the ratio of LFP power at the stimulation frequency (10 ± 1 Hz), to the LFP power across a wide frequency range (3–13 Hz; Fig. 3A). Similar to previous reports,^19^^,^^23^ in pre-epileptic conditions, we found that all individual mice expressing ChR2-mCherry in MSGNs had a highly significant increase in the entrainment power ratio upon optical stimulation when compared to baseline epochs at the intermediate ipsilateral channel (Kolmogorov-Smirnov test, *n*** **=** **120 stimulation epochs per mouse, *n*** **=** **5 mice, *P*** **= 9.19 × 10^−14^, 3.5 × 10^−25^, 2.95 × 10^−11^, 3.61 × 10^−21^ and 3.41 × 10^−43^) (Fig. 3B). We did not detect a shift to higher entrainment values in any individual control mice expressing only mCherry in MSGNs (Kolmogorov-Smirnov test, 120 stimulation epochs per mouse, *n*** **=** **4 mice, *P*** **=** **0.06, 0.26, 0.62, 0.26) (Fig. 3B). We calculated the efficiency of optogenetic pacing by subtracting the baseline entrainment ratio from the entrainment ratio during stimulation at each epoch. The median entrainment efficiency was significantly higher across all electrode locations in mice expressing ChR2-mCherry when compared to mCherry control mice (two-way ANOVA, Tukey *post hoc* test, *P*** **=** **0.013, 0.020, 0.008, 0.049, 0.005 for intermediate-ipsilateral, caudal-ipsilateral, rostral-contralateral, intermediate-contralateral, caudal-contralateral electrode locations, respectively; df = 4, F = 0.18, *n*** **=** **120 trials per mouse, *n*** **=** **5 mice) (Fig. 3C). Together, the PLV and entrainment analyses indicate that MSGN rhythmic optogenetic stimulation is capable of pacing oscillations bilaterally throughout the rostral to caudal extent of the hippocampus.

We next used PLV analysis to quantify whether MSGN activation effectively modulates hippocampal activity in conditions of chronic epilepsy with hippocampal sclerosis. We injected kainate through the previously implanted cannula and mice were recorded 3 weeks after injection to allow for the establishment of hippocampal sclerosis, which we confirmed in *post hoc* anatomical analysis (Supplementary Fig. 3A). We again stimulated MSGNs with 10 Hz medial septal optical stimulation. We found that hippocampal sclerosis had no obvious effect on modulation of hippocampal oscillations by optogenetic stimulation of MSGNs across electrodes (Supplementary Fig. 4) including at the intermediate-ipsilateral location, where seizures are frequently recorded in the intrahippocampal kainate TLE model.^5^^,^^47^ We also found no change in PLVs when compared to pre-epileptic conditions (two-way repeated measures ANOVA, df = 4, F = 0.40498, *P*** **=** **0.55912, *n*** **=** **120 epochs per mouse, *n*** **=** **5 mice) (Fig. 2G). Furthermore, the entrainment efficiency of MSGN rhythmic optical stimulation over hippocampal oscillations was not significantly reduced in any electrode locations when comparing epileptic to baseline conditions (two-way repeated measures ANOVA, df = 4, F = 0.01, *P*** **=** **0.91 Tukey *post hoc* test, *n*** **=** **120 baseline and stimulation epochs per condition, *n*** **=** **5 mice) (Fig. 3D).

**Figure 4:**
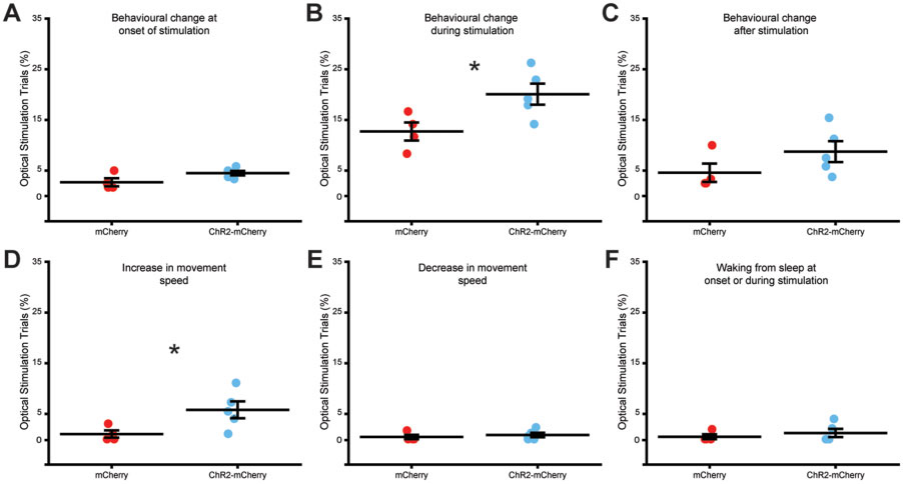
**Behavioural effects of MSGN optical stimulation.** (**A**) Plot of percentage of optical stimulation trials in which a behavioural change occurs at stimulation onset in mice expressing mCherry or ChR2-mCherry in MSGNs. Horizontal lines indicate mean values (mean ± SEM). Points correspond to percentage from individual mice. There was no significant difference between mCherry and ChR2-mCherry expressing mice (two-sample *t*-test two-sided, df = 7, T = −2.09, *P *=* *0.07, *n *=* *4 mCherry and 5 mCherry-ChR2). (**B**) Plot of percentage of optical stimulation trials in which a behavioural change occurs throughout the trial in mice expressing mCherry or ChR2-mCherry in MSGNs. Horizontal lines indicate mean values (mean ± SEM). Points correspond to percentage from individual mice. ChR2-mCherry expressing mice had a significantly higher percentage (two-sample *t*-test two-sided, df = 7, T = −2.61, *P *=* *0.03, *n *=* *4 mCherry and 5 mCherry-ChR2). (**C**) Plot of percentage of optical stimulation trials in which a behavioural change occurs at the end of stimulation in mice expressing mCherry or ChR2-mCherry in MSGNs. Horizontal lines indicate mean values (mean ± SEM). Points correspond to percentage from individual mice. There was no significant difference between mCherry and ChR2-mCherry expressing mice (two-sample *t*-test two-sided, df = 7, T = −1.47, *P *=* *0.19, *n *=* *4 mCherry and 5 mCherry-ChR2). (**D**) Plot of percentage of optical stimulation trials in which there is an increase in movement speed throughout the trial in mice expressing mCherry or ChR2-mCherry in MSGNs. Horizontal lines indicate mean values (mean ± SEM). Points correspond to percentage from individual mice. ChR2-mCherry expressing mice had a significantly higher percentage (two-sample *t*-test two-sided, df = 7, T = −2.39, *P *=* *0.04, *n *=* *4 mCherry and 5 mCherry-ChR2). (**E**) Plot of percentage of optical stimulation trials in which there is a decrease in movement speed throughout the trial in mice expressing mCherry or ChR2-mCherry in MSGNs. Horizontal lines indicate mean values (mean ± SEM). Points correspond to percentage from individual mice. There was no significant difference between mCherry and ChR2-mCherry expressing mice (two-sample *t*-test two-sided, df = 7, T = −0.63, *P *=* *0.55, *n *=* *4 mCherry and 5 mCherry-ChR2). (**F**) Plot of percentage of optical stimulation trials in which there is an increase in behavioural speed throughout the trial in mice expressing mCherry or ChR2-mCherry in MSGNs. Horizontal lines indicate mean values (mean ± SEM). Points correspond to percentage from individual mice. ChR2-mCherry expressing mice had a significantly higher percentage (two-sample *t*-test two-sided, df = 7, T = −0.73, *P *=* *0.05, *n *=* *4 mCherry and 5 mCherry-ChR2).

We performed manual video analysis to assess whether MSGN optical stimulation is associated with adverse behavioural effects. We did not record instances of spasms or motor seizures upon stimulation in mCherry-only or ChR2-mCherry expressing animals in pre-epileptic or chronically epileptic conditions. Across the multiple behaviours we analysed including grooming, eating, exploring, quiet rest and sleep, we saw changes in <21% of stimulation trials (Fig. 4 and Supplementary Fig. 5). There was no significant difference in the percentage of stimulation epochs between mCherry and ChR2-mCherry expressing animals, counting both pre-epileptic and epileptic conditions, in changes in behaviour at the onset (two-sample *t*-test two-sided, df = 7, T = −2.09, *P*** **=** **0.07, *n*** **=** **4 mCherry and 5 mCherry-ChR2; Fig. 4A) or at the end of stimulation (two-sample *t*-test two-sided, df = 7, T = −1.47, *P*** **=** **0.19, *n*** **=** **4 mCherry and 5 mCherry-ChR2; Fig. 4C). There was a 7.4% increase in the percentage of trials with a change of ongoing behaviour throughout the duration of stimulation in mCherry-ChR2 expressing animals (two-sample *t*-test two-sided, df = 7, T = −2.61, *P*** **=** **0.03, *n*** **=** **4 mCherry and 5 mCherry-ChR2; Fig. 4B), although this was still in a minority of trials. Similarly, there was an increase in the speed of the ongoing movement throughout the stimulation in mCherry-ChR2 expressing mice, albeit only in 5.8% of the trials (two-sample *t*-test two-sided, df = 7, T = −2.39, *P*** **=** **0.04, *n*** **=** **4 mCherry and 5 mCherry-ChR2; Fig. 4D). There was no difference between mCherry and ChR2-mCherry animals in the percentage of stimulations in which a movement’s speed decreased (two-sample *t*-test two-sided, df = 7, T = −0.63, *P*** **=** **0.55, *n*** **=** **4 mCherry and 5 mCherry-ChR2; Fig. 4E) or in the number of times an animal woke from sleep throughout the stimulation (two-sample *t*-test two-sided, df = 7, T = −0.73, *P*** **=** **0.05, *n*** **=** **4 mCherry and 5 mCherry-ChR2; Fig. 4F). There were no significant differences in any behavioural measures between pre-epilepsy and chronic epilepsy conditions in ChR2-mCherry expressing animals (paired *t*-tests two-sided, df = 4, T = 2.44, 2.12, −0.30, 2.53, 1.80 and −1, *P*** **=** **0.07, 0.10, 0.78, 0.06, 1.81 and 0.37 for behaviour change at onset, change during, change at end, speed increase, speed decrease and wake from sleep, respectively) (Supplementary Fig. 5). These data suggest that the adverse behavioural effects of MSGN optical stimulation are minimal.

Together, these results demonstrate that MSGNs remain functional despite hippocampal sclerosis in conditions of chronic TLE and can modulate hippocampal LFP oscillations with minor adverse effects on behaviour. As such, stimulation of MSGNs may be able to disrupt ongoing epileptic seizures.

### Decrease in seizure duration upon wireless closed-loop stimulation of MSGNs

**Figure 5:**
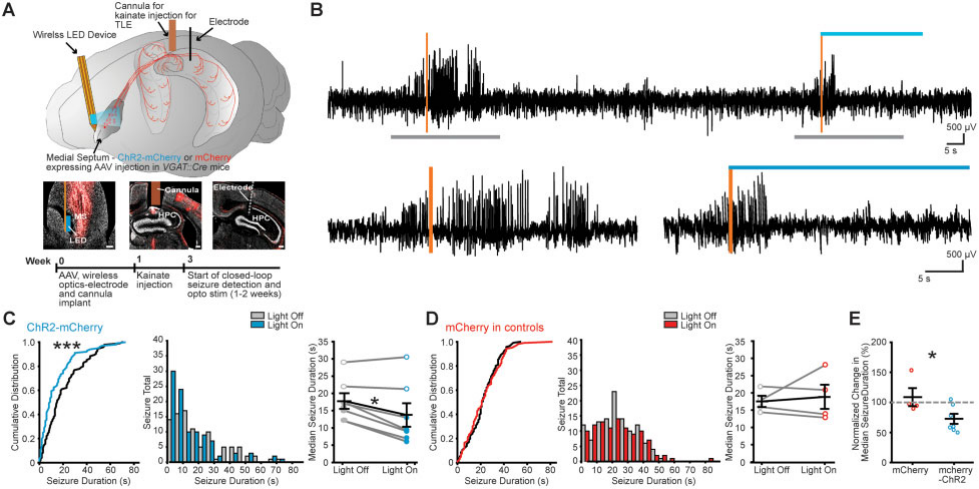
**Wireless closed-loop rhythmic optical MSGN stimulation reduces spontaneous seizure duration in chronic epilepsy.** (**A**) *Top*: Schematic of viral expression and implantation of wireless-LED device and LFP electrode. Cre-dependent ChR2-mCherry or mCherry were expressed in MSGNs by AAV injection into the medial septum of *VGAT::cre* transgenic mice. A wireless optogenetic device was implanted lateral to the medial septum. A cannula for kainate injection and LFP electrode were implanted in the hippocampus, connected to a wireless electrophysiology transmitter located subcutaneously over the back of the mice. Closed-loop seizure identification began at least 2 weeks after kainate injection and establishment of chronic seizures. *Middle*: Neurotrace (grey) labelled sections and ChR2-mCherry (red) expressed in the medial septum (MS) and in MSGN axons in the hippocampus (HPC) including locations of the optical fibre (*left*), the cannula (*middle*) and the electrode track (*right*). Scale bars = 100 µm. *Bottom*: Experimental timeline. (**B**) *Top*: Example LFP trace during detection of electrographic seizures (vertical orange bars), activating the wireless LED (blue horizontal bar) for 30 s randomly in 50% of detected seizures. *Bottom*: Expanded time over grey bars in *top*. (**C** and **D**) Light off and light on (10 Hz stimulation) in individual ChR2-mCherry (**C**) and control mCherry-only (**D**) expressing example mice. Cumulative probability distribution (*left*) and histogram (*middle*) for individual mice (*n *=* *196 and 219 seizures in ChR2-mcherry and mCherry expressing mice, respectively) (***P <* *0.002; Kolmogorov-Smirnov test, two-sided). Plot of light on and light off group median seizure durations (*right*). Horizontal lines indicate mean values (mean ± SEM) and points correspond to median values from individual mice (filled points = *P *<* *0.0001 Kolmogorov-Smirnov test for individually significant mice; *P <* *0.05; paired Wilcoxon signed-rank test, two-sided, across all mice). (**E**) Normalized change in median seizure duration between light off and light on conditions per mouse in mCherry and ChR2-mCherry expressing mice. Horizontal lines indicate mean values (mean ± SEM) and points correspond to median values from individual mice. Rhythmic optical stimulation after seizure detection reduced normalized seizure durations in mice expressing ChR2-mCherry in MSGNs (two-sample *t*-test, two-sided, *n *=* *4 mCherry and 7 mCherry-ChR2 mice, *P <* *0.05).

As in hippocampal-wide LFP modulation experiments, we injected Cre-dependent AAV encoding ChR2-mCherry or mCherry-only in controls in the medial septum of *VGAT::Cre* mice (Fig. 5A). To allow for chronic closed-loop stimulation upon seizure detection to be performed in freely moving mice, we implanted a wireless optogenetic device, equipped with a needle fitted with a micro-LED on the tip, adjacent to the medial septum. We implanted a cannula for unilateral kainate injection over the rostral hippocampus to induce chronic seizures. An LFP electrode targeting the molecular layer of the dentate gyrus in the hippocampus was placed at an intermediate rostral-to-caudal location ipsilateral to the site of kainate injection, where electrographic seizures can frequently be detected^5^^,^^48^ (Fig. 5A, see Supplementary Fig. 6 for confirmed optical device and electrode histological locations). The LFP electrode was connected to a subcutaneous transmitter in the back of each mouse. We performed online electrographic seizure detection using a custom-made algorithm that allowed for accurate and rapid closed-loop functionality (Supplementary material). The program activated the LED on 50% of randomly selected seizures, as in previous closed-loop stimulation studies.^5^^,^^11^^,^^13^^,^^49^

We found that optogenetic stimulation of MSGNs for 30 s at 10 Hz effectively reduced electrographic seizure durations when compared to no stimulation in five of seven mice injected with AAV expressing mCherry-ChR2 (Kolmogorov-Smirnov test two-sided, *P*** **=** **0.002, 0.02, 0.03, 0.045, 0.005, 0.71 and 0.56 for seizure duration comparison in each mouse, *n*** **=** **7 mice with 196, 139, 134, 114, 135, 61 and 37 seizures recorded in each, respectively) (Fig. 5B and C). Furthermore, we found that median seizure durations across the group of mice were significantly shorter upon optical stimulation when compared to no-stimulation (paired Wilcoxon signed-rank test, two-sided, W = 26, *P*** **=** **0.047, *n*** **=** **7 mice; Fig. 5C). In contrast, optical stimulation in control mice expressing only mCherry in MSGNs had no effect on electrographic seizure durations in any of the individual mice tested (Kolmogorov-Smirnov test two-sided, *P*** **=** **0.12, 0.40, 0.39 and 0.57 for seizure duration comparison in each mouse, *n*** **=** **4 mice with 18, 51, 78 and 219 seizures recorded in each, respectively) (Fig. 5D). Similarly, there was no effect on the median seizure duration as a group of mice (paired Wilcoxon signed-rank test two-sided, W = 6, Z = 0.18, *n*** **=** **4 mice, *P*** **=** **0.86; Fig. 5D). Finally, we found that the median change in seizure duration normalized to light off detected seizures was significantly reduced in ChR2-mCherry expressing mice when compared to mCherry expressing controls (two-sample *t*-test two-sided, df = 9, T = 2.4 *P*** **=** **0.04, *n*** **=** **4 mCherry and 7 mCherry-ChR2 expressing mice; Fig 5E).

To test whether seizure blockade has a lasting effect on the epileptic network, as has been reported following activation of cerebellar PV neurons,^13^ we analysed the distribution of intervals between seizures. However, we found that there was no significant change in the median interval following stimulation during a seizure versus when a seizure was not stimulated in ChR2-mCherry expressing animals (paired Wilcoxon signed-rank test two-sided, W = 17, Z = 0.42, *P*** **=** **0.67, *n*** **=** **7 mice; Supplementary Fig. 7A), suggesting that the effects of stimulation are limited to ongoing seizures.

Seizures with behavioural effects are also prevalent in this TLE model.^30^^,^^50^ There was a non-significant trend towards a reduction in median seizure severity upon optogenetic stimulation of MSGNs when compared to no-stimulation seizures (paired *t*-test two-sided, T = 2.36, df = 6, *P* = 0.06, *n*** **=** **7 mice; Supplementary Fig. 7B). We attempted to quantify whether optogenetic stimulation lead to a change in the frequency of tonic-clonic generalized motor seizures, as performed in recent studies,^5^^,^^11^ however the occurrence of these events is low and necessitates recordings over a month in duration to record a sufficient number of seizures. We were limited by the battery of our current wireless transmitters, which do not permit more than 3-week recordings and consequently recorded few tonic-clonic seizures in most animals (Supplementary Fig. 7C).

Together, these results show that MSGN wireless closed-loop optical stimulation can reduce the duration of spontaneous electrographic seizures in the intrahippocampal kainate TLE model with hippocampal sclerosis.

## Discussion

We show that MSGNs and their projections throughout the rostral-to-caudal extent of the hippocampus survive and remain functional as they can be optically stimulated to generate oscillations in a chronic mouse model of TLE with hippocampal sclerosis. Furthermore, we found that wireless closed-loop optogenetic stimulation of MSGNs reduced the duration of spontaneously occurring electrographic seizures. These results reveal a novel potential target for therapy for intractable TLE.

In contrast to a previous study, where MSGNs were found to be vulnerable in a systemic model of TLE,^51^ we found that MSGNs and their projections throughout the rostral-to-caudal extent of the hippocampus remained despite focal hippocampal sclerosis. In previous work assessing MSGN susceptibility to TLE, pilocarpine was administered via intraperitoneal injection.^51^ Muscarinic receptors, which are sensitive to pilocarpine, are expressed by MSGNs^52^ and their activation through systemic administration may result in overexcitability leading to MSGN cell damage. We found that MSGNs and cholinergic populations were not reduced in the chronic intrahippocampal kainate model, which replicates unilateral hippocampal sclerosis, a common feature of intractable TLE,^53–55^ and spontaneously occurring seizures.^30^ Similarly, despite a previous report showing a decrease of connective fibres between the medial septum and hippocampus in patients with TLE with hippocampal sclerosis,^28^ there were no reductions in putative synaptic connections from MSGNs in any hippocampal areas, including the site of kainate injection where there is most sclerotic damage.^42^ The decrease in connective fibres between the medial septum and hippocampus, if replicated in the intrahippocampal kainate model, may reflect the loss of other neuronal types in the medial septum such as glutamatergic cells^56^^,^^57^ or GABAergic neurons that project to the medial septum from the hippocampus.^58–60^

We found that MSGNs, despite hippocampal sclerosis, retained their functionality and were able to modulate the oscillatory activity throughout the rostral-to-caudal extent of the hippocampus with electrodes implanted in the molecular layer of the dentate gyrus. Phase analysis of rhythmic activation of MSGNs showed that LFP timing during stimulation was highly consistent across trials. MSGNs specifically target inhibitory neurons across the hippocampal formation,^16^^,^^18^^,^^40^^,^^61^ and it is hypothesized that both normally-occurring and optically-entrained hippocampal theta oscillations are mediated by MSGNs inducing rebound firing in hippocampal GABAergic neurons, which in turn cause rhythmic firing of principal cells.^16^ However, loss of some hippocampal GABAergic subtypes has been reported in both patients and animal models of TLE.^42^^,^^62^^,^^63^ Therefore, it is possible that even if putative synapses are present, their cellular targets are compromised. The highest level of GABAergic cell loss in the intrahippocampal model of TLE occurs at rostral ipsilateral sites near the injection site, with GABAergic cell loss tapering off between intermediate and caudal locations.^42^ Despite this, we found that the capacity of MSGNs to entrain oscillations in conditions of chronic seizures was not reduced when electrodes are placed in the molecular layer of the dentate gyrus, an area important for controlling the spread of seizures,^11^^,^^44–46^^,^^64^ suggesting that the remaining MSGN connections onto hippocampal GABAergic neurons are able to modulate the oscillatory rhythm at sclerotic locations. Nonetheless, it may be that the level of modulation varies in other hippocampal laminae such as CA1 and CA3 due to microcircuit differences including principal cell function, GABAergic circuitry, and connectivity with external targets,^65^^,^^66^ as well as the level of principal cell death due to hippocampal sclerosis, which is prevalent in CA1 and CA3.^30^

An important component of translatability of a potential cellular target is whether its stimulation results in adverse effects. Previous studies in which parvalbumin positive MSGNs, or their hippocampal terminals, were optogenetically stimulated reported that there was no effect on the animal’s speed of locomotion^23^ or that movements were not induced at rest, but during movement the animal’s speed was slowed.^19^ We found that there was no obvious induction of spasms or motor seizures and only a minority of stimulation epochs resulted in a behavioural change. MSGN stimulation therefore may have a minor effect on motor movements or may indirectly influence glutamatergic medial septal cells, which have been reported to influence locomotion speed.^56^ Furthermore, animals were rarely woken from sleep upon stimulation. Nonetheless, it is unclear whether this type of stimulation would adversely affect cognition. A recent report suggests that pan-neuronal stimulation of medial septal neurons does not perturb active spatial memory.^67^ Additionally, both medial septal electrical stimulation in a chronic TLE rat model^68^ and optogenetic stimulation of parvalbumin MSGNs in an Alzheimer’s disease mouse model,^69^ improve spatial memory deficits.

We report that spontaneous seizures in the intrahippocampal chronic model of epilepsy were detected and acted upon with closed-loop optogenetics in real time through a fully wireless system. Tethered recording conditions can increase stress levels in animals,^70^ potentially leading to increasing seizure susceptibility.^71^ Fully wireless experiments therefore represent a major step forward in animal welfare and accurate modelling of TLE.

We found that 10 Hz optical stimulation, a frequency in the range of normally occurring oscillations as well as LFP spiking activity during prolonged TLE seizures, was able to reduce electrographic seizure durations. We list some possible mechanisms that could result in seizure disruption after rhythmic stimulation of MSGNs: (i) imposing an oscillatory rhythm onto the epileptic network could disrupt seizures by entraining hippocampal GABAergic cells, which would consequently modify their activity and post-action potential refractory periods, as well as that of their principal cell targets. This may prevent cells from reaching threshold during synchronized seizure inputs; (ii) by stimulating with a 10 Hz frequency, which is within the theta range at which the circuit oscillates in physiological conditions, a resynchronization rhythm may compete with the intrinsic synchrony during the seizure and reset the network; (iii) constant rhythmic activation of MSGNs could lead to an excess of GABA in the extracellular space, leading to overall network inhibition and seizure blockade; (iv) MSGNs form monosynaptic connections onto hippocampal GABAergic neurons^15^^,^^16^^,^^18^^,^^40^ and consequently directly inhibit them. It is hypothesized, and has been shown in various epilepsy models^14^^,^^72–75^ and human tissue,^76^ that GABAergic transmission may become excitatory in epileptic conditions due to the reversal of the chloride potential in principal cells after the emergence of excess extracellular potassium. Consequently, directly inhibiting hippocampal GABAergic neurons through MSGN activation may lead to a paradoxical decrease in excitation during seizures; and (v) MSGNs also project to the subiculum and medial entorhinal cortex,^16^^,^^77^ structures implicated in seizure generation and spread.^14^^,^^64^ Thus, blockade of seizures may involve modulation of additional structures outside the hippocampus or hippocampal output structures.

Our results contrast with a previous study showing that in kindled animals, cholinergic medial septal neuron stimulation reduces seizure occurrence and severity, while MSGN stimulation has no effect.^28^ This divergence could be due to differences in how seizures are generated and the stimulation protocol in each study. Here, stimulation occurs upon spontaneous seizure detection once the network is epileptic after an initial chemical insult and refractory period. In the previous study,^28^ seizures are generated upon successive electrical insults with optical MSGN stimulation occurring immediately after each insult. Additionally, cholinergic and GABAergic medial septal neurons interact within the medial septum^78^ and co-release of acetylcholine and GABA from medial septal terminals may occur in the hippocampus.^79^ Therefore, stimulation of one population of neurons may modulate the other, or both neurotransmitters may be released upon stimulation of either population, consequently resulting in non-specific effects. Indeed, while our experiments establish a proof-of-principle for the effectiveness of closed-loop optical stimulation of MSGNs, non-selective activation of medial septum projections may be sufficient to modulate hippocampal oscillations^67^^,^^80^ and reduce seizure frequencies.^81^

It remains unclear which patterns and frequencies of MSGN stimulation are most effective at controlling seizures. Stimulation of MSGN terminals in the hippocampus entrains oscillations in non-epileptic animals more effectively in the theta range (6–12 Hz) than at frequencies outside of that range (2, 4 and 20 Hz).^19^ We found that closed-loop 10 Hz MSGN stimulation reduced electrographic seizure durations. A recent preprint suggests that stimulation of MSGNs that precisely matches individual seizure LFP voltage deflections can block seizures in a rat kindling model.^82^ Future work comparing stimulation parameters, including multiple frequencies and whether to apply stimulation continuously or in a closed-loop manner, across TLE models and potential cellular and anatomical targets will improve our understanding of the effectiveness and translatability of these potential treatment strategies.

Our study highlights MSGNs as a potential new target to treat TLE with hippocampal sclerosis and may prompt the development of novel gene therapy or deep brain stimulation strategies to test the efficacy of this population in treating patients with intractable seizures.

## Supplementary Material

awab042_Supplementary_Data

## Abbreviations

AAV: adeno-associated virus
LFP: local field potential
MSGN: medial septal GABAergic neuron
PLV: phase locking value
TLE: temporal lobe epilepsy
