# Erratum to: Medial septal GABAergic neurons reduce seizure duration upon optogenetic closed-loop stimulation

**DOI:** 10.1093/brain/awab212

**Published:** 2021-06-25

Katerina Hristova, Cristina Martinez-Gonzalez, Thomas C. Watson, Neela K. Codadu, Kevan Hashemi, Peter C. Kind, Matthew F. Nolan and Alfredo Gonzalez-Sulser. Medial septal GABAergic neurons reduce seizure duration upon optogenetic closed-loop stimulation. *Brain*. 2021;144(5):1576-1589. doi:10.1093/brain/awab042

The publisher apologizes for publishing an incorrect version of the article. This has been corrected.

