# Supplementary material for: Medial septal GABAergic neurons reduce seizure duration upon optogenetic closed-loop stimulation
Source: Brain. 2021 Mar 26;144(5):1576–89. doi: 10.1093/brain/awab042 (PMC8219369; doi:10.1093/brain/awab042)
Supplement: awab042_Supplementary_Data [file awab042_supplementary_data.pdf]

Supplementary Information

Supplementary Table 1. List of primary antibodies used for immunofluorescence labelling.

Antigen	*Supplier*	*Catalogue No.*	*RRID No.*	*Host species*	*Dilution*
GFP	Abcam	AB13970	AB_300798	Chicken	1:500
mCherry	Invitrogen	M11217	AB_2536611	Rat	1:1000
Parvalbumin	Swant	PV235	AB_10000343	Mouse	1:2000
Calbindin	Swant	CB38	AB_2721225	Rabbit	1:1000
ChAT	Merck Millipore	AB144P	AB_2079751	Goat	1:500
GABA	Sigma	A2052	AB_477652	Rabbit	1:1000

Supplementary Table 2. List of secondary antibodies used for immunofluorescence labelling.

Secondary antibodies (Thermofisher Scientific)	*Catalogue No.*	*Target species*	*Dilution*
Alexa Fluor 488 Goat anti-Chicken IgY (H+L)	A-11039	Chicken	1:500
Alexa Fluor 546 Goat anti-Rat IgG (H+L)	A-11081	Rat	1:500
Alexa Fluor 647 Goat anti-Mouse IgG (H+L)	A-21236	Mouse	1:500
Alexa Fluor 555 Goat anti-Mouse IgG (H+L)	A-21422	Mouse	1:500
Alexa Fluor 488 Goat anti-Mouse IgG (H+L)	A-11001	Mouse	1:500
Alexa Fluor 405 Goat anti-Mouse IgG (H+L)	A-31553	Mouse	1:250
Alexa Fluor 647 Goat anti-Rabbit IgG (H+L)	A-21244	Rabbit	1:500
Alexa Fluor 546 Goat anti-Rabbit IgG (H+L)	A-11010	Rabbit	1:500
Alexa Fluor 405 Goat anti-Rabbit IgG (H+L)	A-31556	Rabbit	1:250
Alexa Fluor 647 Donkey anti-Goat IgG (H+L)	A-21447	Goat	1:1000
Alexa Fluor 546 Donkey anti-Goat IgG (H+L)	A-11056	Goat	1:1000
Alexa Fluor 555 Donkey anti-Mouse IgG (H+L)	A-31570	Mouse	1:1000
Alexa Fluor 488 Donkey anti-Mouse IgG (H+L)	A-21202	Mouse	1:1000
Alexa Fluor 350 Donkey anti-Rabbit IgG (H+L)	A10039	Rabbit	1:250

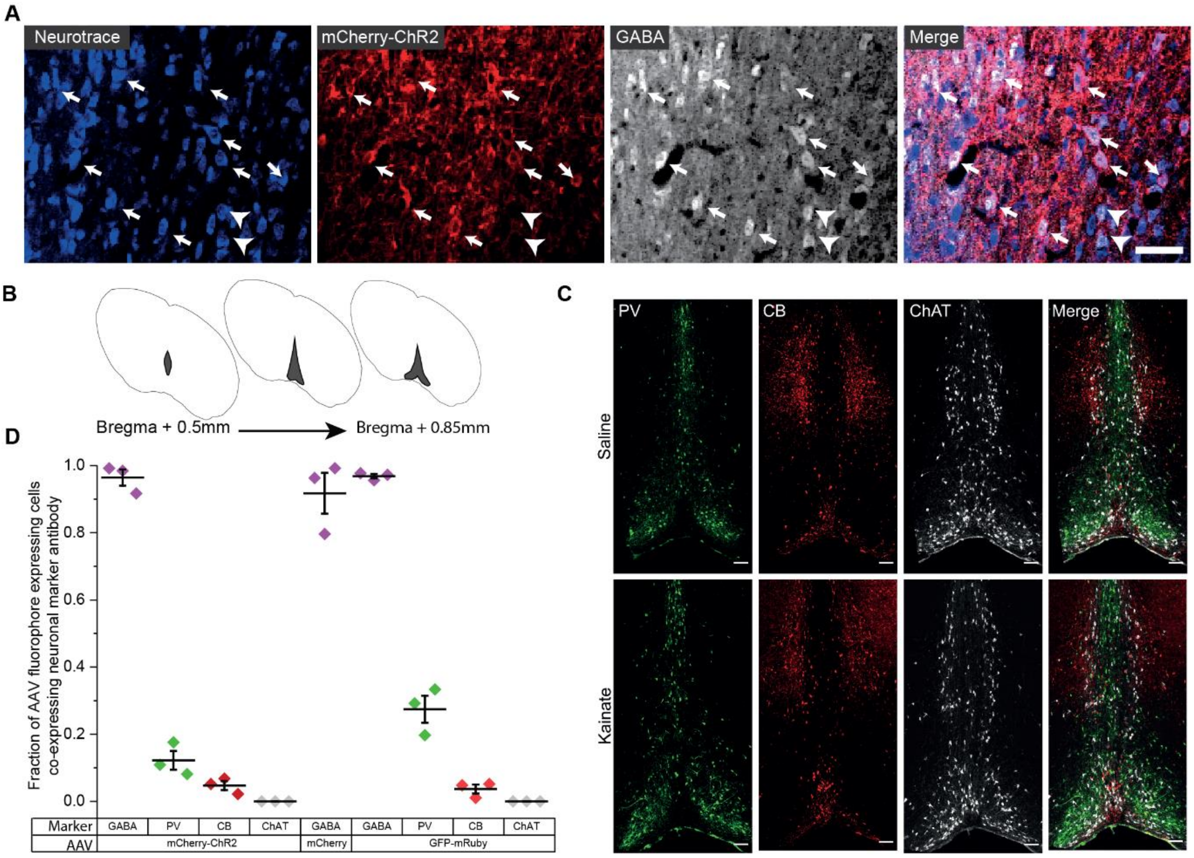

Figure S1 – Specificity of AAV expression to MSGNs and resilience of medial septal neurons to hippocampal sclerosis

A) Representative mCherry-ChR2 expression in VGAT MSGNs and immunohistochemical staining for Neurotrace and GABA. Note: examples of neurons expressing GABA and VGAT mCherry-ChR2 (arrows) and neurons expressing GABA but not VGAT (arrowheads). Scalebar = 50 µm.

B) Schematic of quantification locations of neuronal populations in the medial septum. The total number of cells were counted in each caudal to rostral location (grey area) from bregma for all cell types in each animal.

C) Immunohistochemical staining of neuronal markers for parvalbumin (PV), calbindin (CB) and choline acetyltransferase cells (ChAT) in saline (top) and kainate (bottom) treated mice. Scalebars = 100 µm.

D) Fraction of AAV fluorophore expressing cells co-expressing antibodies across neuronal population markers. Horizontal lines indicate mean values (mean ± SEM). Points correspond to values from individual mice (n= 3 mice per population). Note: high level of co-expression of AAVs fluorophores with GABA stained neurons.

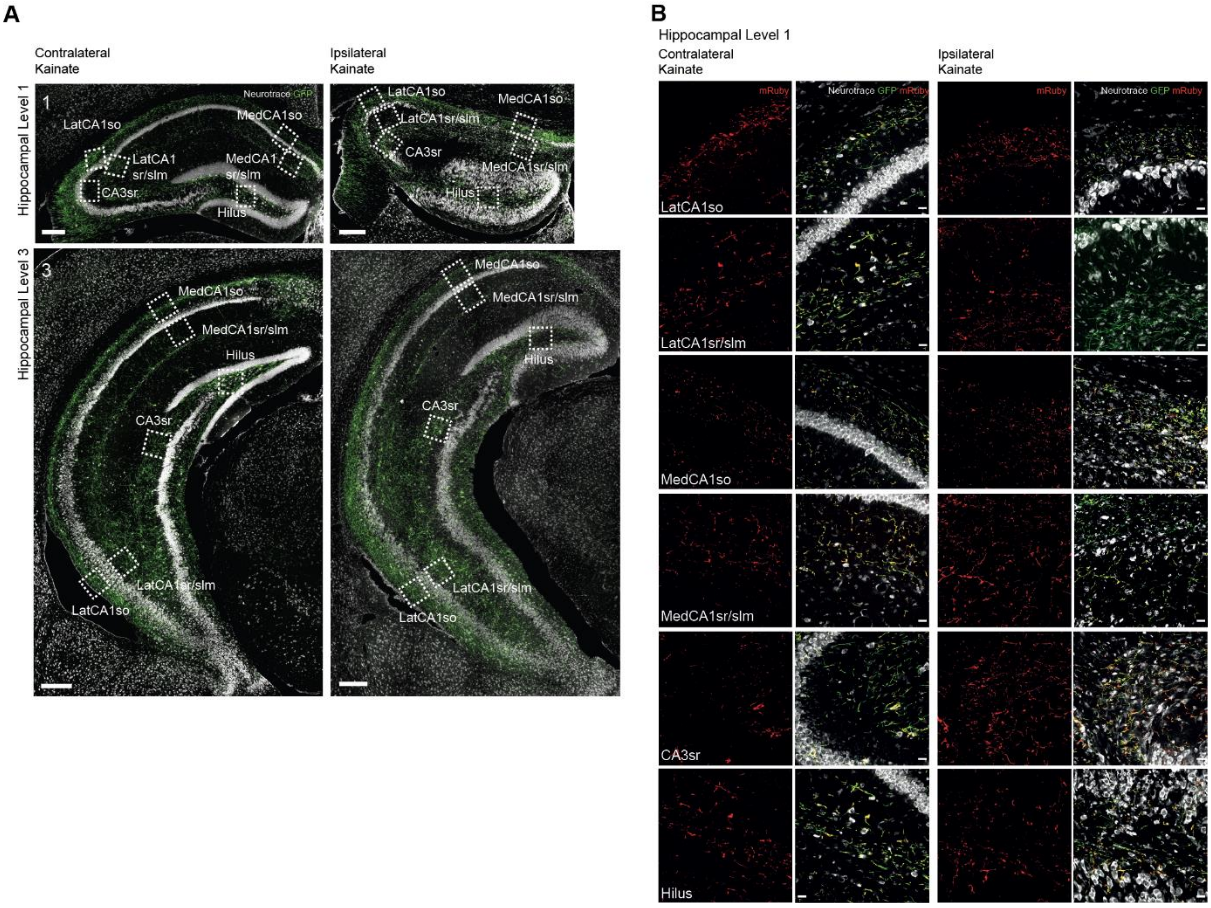

Figure S2 – Imaging of putative synapses across the hippocampus

A) Representative locations of high-resolution imaging across hippocampal sections. Rostral to caudal hippocampal levels 1 and 3 of a kainate-injected mouse expressing mRuby-Synaptophysin-GFP in MSGNs with staining by fluorescent Neurotrace are shown for the contralateral and ipsilateral hippocampi with labelled locations of high resolution mRuby puncta imaging (square boxes). Scalebars = 200 µm. Note: expression of GFP (green) in MSGN axons across the hippocampus and sclerosis in rostral level ipsilateral to kainate injection.

B) Representative 40x images of areas imaged across the hippocampus for mRuby puncta analysis at rostral-caudal level 1. Scalebars = 10 µm. *Lat* = lateral, *Med* = medial, *so* = stratum oriens, *sr* = stratum radiatum, *slm* = stratum lacunosum moleculare.

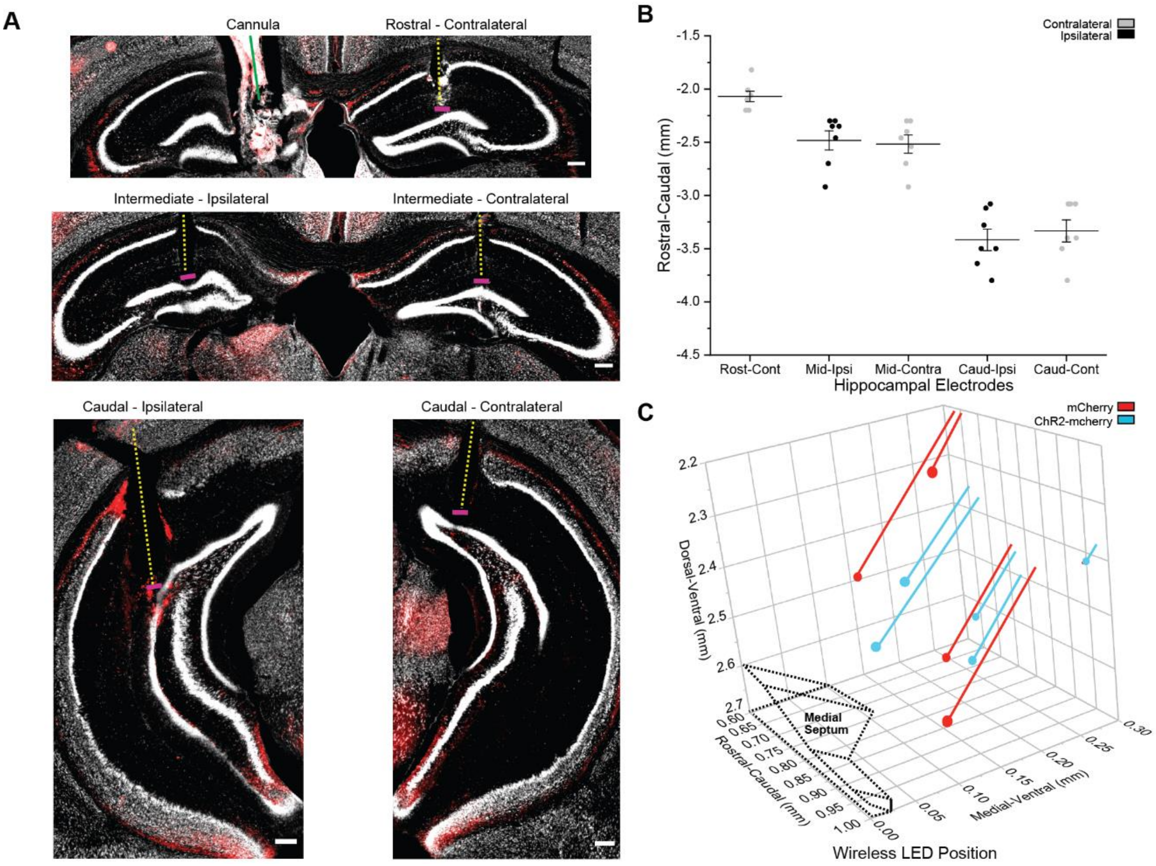

Figure S3 – Histology of tethered entrainment recordings across the hippocampus.

A) Cannula and anatomical locations of five electrode pair locations across bilateral hippocampi for example mouse. Demarcations for cannula and kainate injection lesion (green line), electrode tracks (yellow dashed line) and final electrode pair positions (magenta lines). Scale bars = 100 µm.

B) Plot of rostral-caudal positions relative to bregma of electrode pair implantations. Horizontal lines indicate mean values for mCherry and ChR2-Cherry expressing mice (mean ± SEM, n =4 and 5 respectively). Points correspond to values from individual mice. Note: all electrode pairs were confirmed to be located at a final depth within lacunosum-moleculare, the molecular or the granule cell layers of the dentate gyrus.

C) Three-dimensional plot of optical fibre final coordinates relative to bregma. Black-dashed lines denotate approximate coordinates of dorsal medial septum. Red and blue lines indicate confirmed optical fibre tips and approximate trajectories for mCherry and ChR2-mCherry expressing mice locations.

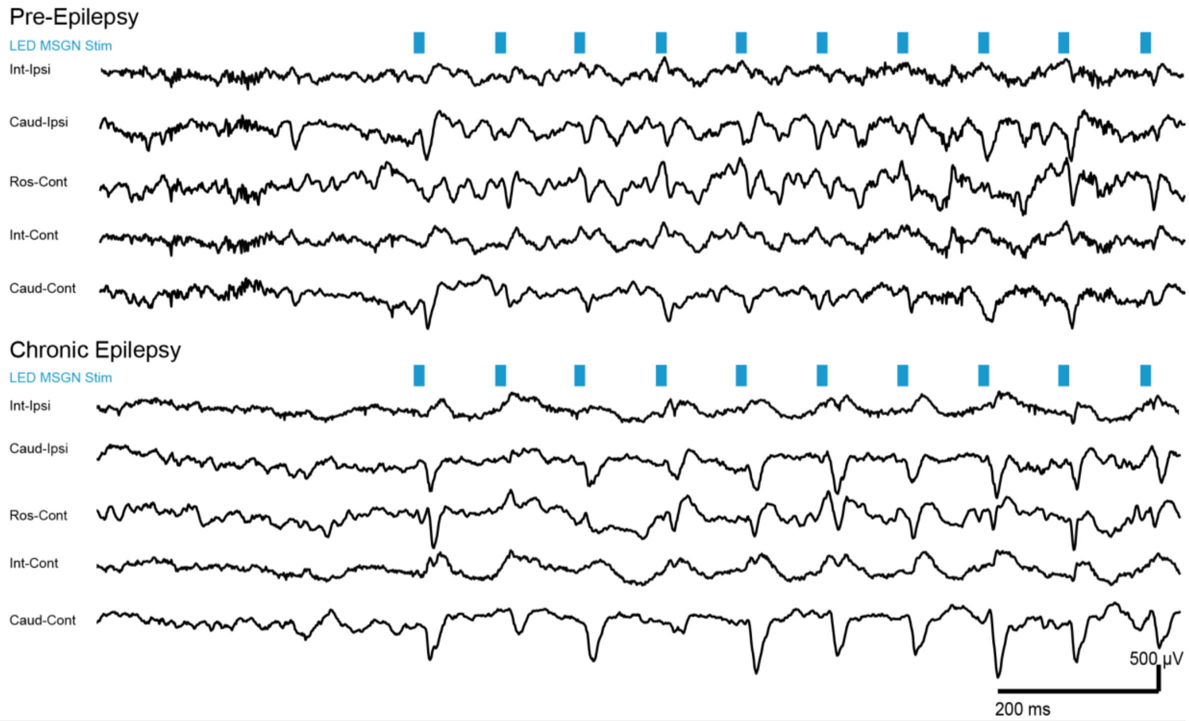

Figure S4 – Entrainment of oscillations across the hippocampus by optical stimulation of MSGNs is not compromised in the presence of hippocampal sclerosis

Representative LFP traces from a mouse in pre-epileptic (top) and chronically epileptic (bottom) conditions from hippocampal channels - rostral contralateral to cannula (Ros-Cont) and bilaterally at intermediate (Int-Ipsi and Int-Cont) and caudal (Caud-Ipsi and Caud-Cont) locations before and after onset of 10 Hz theta optical MSGN stimulation.

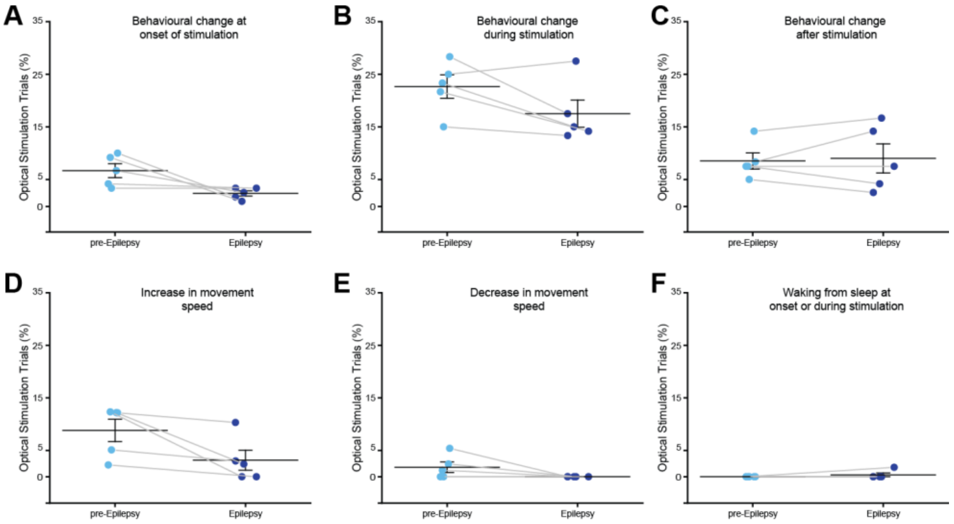

Fig. S5 Behavioural effects of MSGN optical stimulation in pre-epilepsy and chronic epilepsy conditions

A) Plot of percentage of optical stimulation trials in which a behavioural change occurs at stimulation onset in mice expressing ChR2-mCherry in MSGNs in pre-epilepsy and epilepsy conditions. Horizontal lines indicate mean values (mean ± SEM). There was no significant difference between conditions (Paired T-test two-sided, DF = 4, T = 2.44, p = 0.07, n = 5).

B) Plot of percentage of optical stimulation trials in which a behavioural change occurs at stimulation onset in mice expressing ChR2-mCherry in MSGNs in pre-epilepsy and epilepsy conditions. Horizontal lines indicate mean values (mean ± SEM). There was no significant difference between conditions (Paired T-test two-sided, DF = 4, T = 2.12, p = 0.10, n = 5).

C) Plot of percentage of optical stimulation trials in which a behavioural change occurs at stimulation onset in mice expressing ChR2-mCherry in MSGNs in pre-epilepsy and epilepsy conditions. Horizontal lines indicate mean values (mean ± SEM). There was no significant difference between conditions (Paired T-test two-sided, DF = 4, T = -0.30, p = 0.78, n = 5).

D) Plot of percentage of optical stimulation trials in which there is an increase in movement speed at stimulation onset in mice expressing ChR2-mCherry in MSGNs in pre-epilepsy and epilepsy conditions. Horizontal lines indicate mean values (mean ± SEM). There was no significant difference between conditions (Paired T-test two-sided, DF = 4, T = 2.53, p = 0.06, n = 5).

E) Plot of percentage of optical stimulation trials in which there is a decrease in movement speed at stimulation onset in mice expressing ChR2-mCherry in MSGNs in pre-epilepsy and epilepsy conditions. Horizontal lines indicate mean values (mean ± SEM). There was no significant difference between conditions (Paired T-test two-sided, DF = 4, T = 1.80, p = 1.81, n = 5).

F) Plot of percentage of optical stimulation trials in which a behavioural change occurs at stimulation onset in mice expressing ChR2-mCherry in MSGNs in pre-epilepsy and epilepsy conditions. Horizontal lines indicate mean values (mean ± SEM). There was no significant difference between conditions (Paired T-test two-sided, DF = 4, T = -1.00, p = 0.37, n = 5)

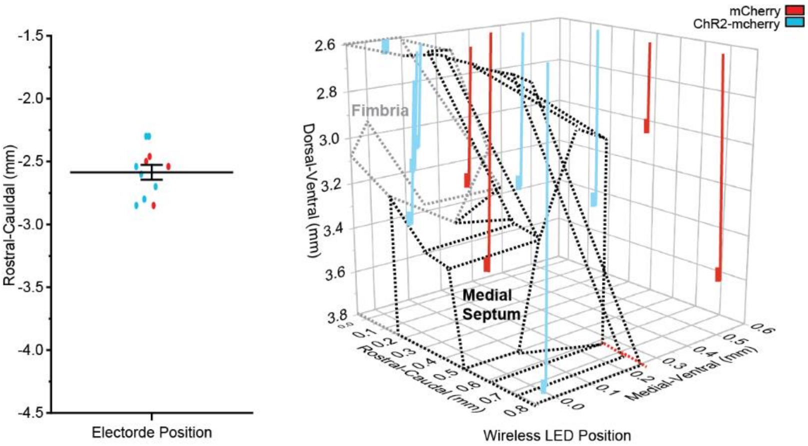

Figure S6 – Histology of wireless optogenetics experiments

Plot of rostral-caudal electrode positions relative to bregma of electrode implantations (left). Horizontal lines indicate mean values for seven ChR2-Cherry (blue) and four mCherry (red) expressing mice (mean ± SEM). Points correspond to values from individual mice. Note: all electrode final locations were confirmed to be located at a final depth within laculosum-moleculare, the molecular or the granule cell layers of the dentate gyrus. Three-dimensional plot of optical fibre final coordinates relative to bregma (right). Dashed lines denotate approximate coordinates of the medial septum (black) and fimbria (grey). Note: projections from MSGNs to the hippocampus travel through the fimbria. Lines indicate approximate wireless optical device needle trajectories and rectangles represent confirmed final LED positions in mCherry (red) and ChR2-mCherry (blue) expressing mice.

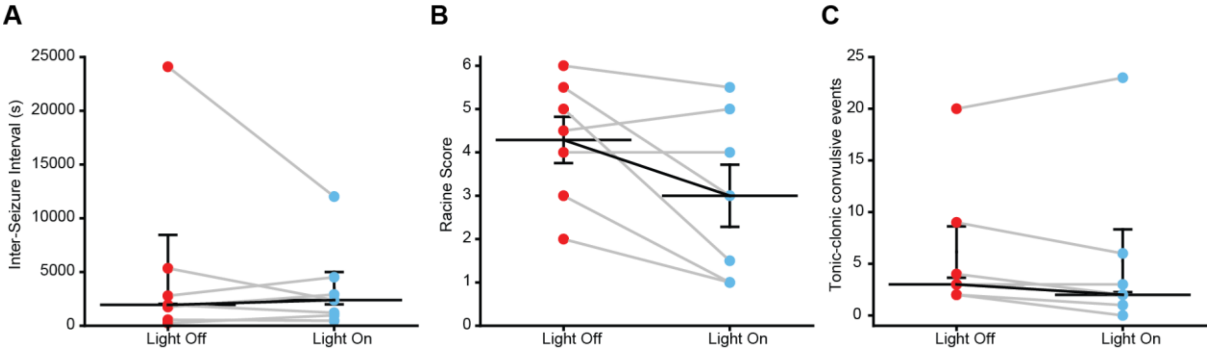

Fig. S7 Effects of MSGN optical stimulation on Inter-Seizure Intervals and Behavioural Seizures

A) Median inter-seizure interval in mice expressing ChR2-mCherry in MSGNs in light off and light on (10 Hz stimulation). Horizontal lines indicate median values (median ± SEM). There was no significant difference between conditions (Paired Wilcoxon Signed Ranks test two-sided, W = 17, Z = 0.42, n = 7 mice, p = 0.67, n = 7 mice).

B) Median Racine score in mice expressing ChR2-mCherry in MSGNs in light off and light on (10 Hz stimulation). Horizontal lines indicate mean values (mean ± SEM). There was no significant difference between conditions (Paired T-test two-sided, T = 2.36, DF = 6, P = 0.06, n = 7 mice).

B) Number of tonic-clonic convulsive events in mice expressing ChR2-mCherry in MSGNs in light off and light on (10 Hz stimulation). Horizontal lines indicate median values (median ± SEM). There was no significant difference between conditions (Paired Wilcoxon Signed Ranks test two-sided, W = 15.5, Z = 0.95, p = 0.34, n = 7 mice).

Supplementary Note 1 - Validation of Seizure Detection Algorithm

The seizure detection algorithm was validated on 93 hours of data recordings from 4 chronically epileptic animals. We performed a detailed analysis on the performance.

**Animal**	**Total Seizures Recorded**	**False Positives**	**False Negatives**	**False Positive (%)**	**False Negative (%)**
1	474	11	2	2.3	0.4
2	33	4	1	12.1	2.94
3	6	0	0	0	0
4	9	0	2	0	18.18

Average seizure detection rate: 0.35 ± 0.31 per hour

Average false positive rate: 0.01 ± 0.007 per hour

Average false negative rate: 0.0033 ± 0.0013 per hour
